# Impact of COVID-19 Lockdown Measures on Spanish People with Chronic Pain: An Online Study Survey

**DOI:** 10.3390/jcm9113558

**Published:** 2020-11-05

**Authors:** Rubén Nieto, Rebeca Pardo, Beatriz Sora, Albert Feliu-Soler, Juan V. Luciano

**Affiliations:** 1 eHealth Lab Research Group, Faculty of Health Sciences, Universitat Oberta de Catalunya, 08018 Barcelona, Spain; 2 Centro de Psicología Aplicada, Universidad Autónoma de Madrid, 28049 Madrid, Spain; rebeca.pardocebrian@gmail.com; 3 Facultad de Ciencias Biológicas, Universidad Europea de Madrid, 28670 Villaviciosa de Odón, Spain; 4 eHealth Lab Research Group, Faculty of Psychology and Education, Universitat Oberta de Catalunya, 08018 Barcelona, Spain; beatriz.sora@urv.cat; 5 Faculty of Educational Sciencies and Psychology, Psychology Department, Universitat Rovira i Virgili, 43007 Tarragona, Spain; 6 Parc Sanitari Sant Joan de Déu, 08830 Sant Boi de Llobregat, Spain; albert.feliu@uab.cat (A.F.-S.); jvluciano@pssjd.org (J.V.L.); 7 Faculty of Psychology, Universitat Autònoma de Barcelona, 08193 Barcelona, Spain

**Keywords:** COVID-19, chronic pain, lockdown, triggers, coping, well-being

## Abstract

The corona virus disease 2019 (COVID-19) pandemic is one of the most important healthcare and societal challenges to have emerged in the last century. It may have effects on both physical and psychosocial health, but studies considering the impact on vulnerable populations, such as people with chronic pain, are needed. In this cross-sectional study, an online survey of relevant chronic pain domains, coping strategies, triggers and potential related variables was answered by 502 Spanish individuals with chronic pain. Participants were mainly women (88%) with longstanding chronic pain and moderate to high pain intensity and disability. The perception of pain aggravation and the most pain-related outcomes were observed. Contextual variables such as job insecurity, worries about the future, people cohabiting, being close to someone who had passed away, or being potentially infected with COVID-19 were related to worse outcomes. More than half the participants altered their pain management style (e.g., increased medication intake) and several changes occurred with respect to pain triggers (cognitions, feelings of insecurity and loneliness, and sleeping problems were more frequently reported as triggers during lockdown). Our preliminary results highlight the negative effects of lockdown on patients with chronic pain as well as the need to make available cost-effective and remotely accessible healthcare resources for counteracting them.

## 1. Introduction

The corona virus disease 2019 (COVID-19) pandemic is one of the most important worldwide healthcare and societal challenges to have emerged in the last century, and it has had dramatic consequences on the population. Providing exact data on the number of infected people and mortality rates is complicated since the health crisis is not yet under control and the death toll is growing daily. For example, in mid-July 2020, there were more than 13,000,000 cases and 574,464 deaths reported to the World Health Organization [1].

Available data on the effects of the crisis and lockdown measures on people’s quality of life and wellbeing remain scant. Wang et al. [2] conducted a study in mainland China in the initial phase of the pandemic and found that 53.8% of the people surveyed (*n* = 1200) reported a moderate to severe psychological impact. There are also reports suggesting an increase in anxiety, depression and stress [3], as well as a rise in suicide rates [4]. Prior pandemics and lockdowns also provide insight into how much of an impact a situation like this can have in these areas. For example, Hawryluck et al. [5] found a high prevalence of depression and post-traumatic stress related to the quarantine brought on by severe acute respiratory syndrome (SARS). The European Foundation for the Improvement of Living and Working Conditions [6] conducted an online survey exploring the effects of COVID-19 in different regards. Although final results have yet to be published, in their preliminary April 2020 report they observed an overall decrease in people’s reported well-being (lowered life satisfaction, happiness, optimism and mental well-being).

Apart from its detrimental impact on health, the pandemic is jeopardizing the worldwide economy, too. For example, there has been an increase in the worldwide unemployment rate [7]. In the United States, it was estimated that 20 million jobs were lost in April [8]. In a preliminary report by the Eurofound [6], 5% of respondents reported losing their jobs permanently and 23%, temporarily. Moreover, 38% of people reported that their financial situation was worse than before the pandemic. In turn, unemployment and job uncertainty have negative effects on individuals’ and communities’ psychological and social well-being [9].

Besides these few studies, there is little research on how people living through the COVID-19 crisis or those having been infected with the disease perceive and experience the situation [10]. Of particular interest for exploration are the views and experiences of people with chronic pain, as COVID-19 can have a greater impact on them [11], as well as on individuals with physical disabilities in general [3]. This is because, among other factors, chronic pain is more frequent in older people and is strongly related to disability, and access to pain management facilities has been disrupted as a consequence of lockdown, to prevent the risk of spreading the COVID-19 disease [11,12]. Chronic pain management difficulties can increase pain, pain-related disability and psychological issues such as depression [13]. In this regard, international panels of experts have recently highlighted the importance of ensuring continued pain treatment, and promote telemedicine from a biopsychosocial approach [14].

It is also important to highlight that some of the reported consequences of the pandemic and resulting lockdown can be triggers of pain. We are referring to factors such as stress. As previously stated, available data suggest that stress increases as a consequence of the pandemic and lockdown. The link between stress and chronic pain has been widely studied in recent decades. For example, we already know that exposure to certain stressful situations increases the risk of developing chronic pain [15]. General anxiety levels and general stress have also been related in general with a poorer adjustment to chronic pain [16,17]. Social isolation, which might have increased for some people as a consequence of lockdown, has a well-known impact on pain [18,19,20]. Similarly, lack of exercise or lack of activity in general can worsen functioning in people with pain [21]. Likewise, sleep problems have also been described during lockdown, and have been related to higher levels of intolerance to uncertainty, concern about COVID-19, loneliness, and severe depressive symptoms [22]. The relationship between sleep disturbances and pain is bidirectional (as is the relationship between stress and pain), so that pain disturbs sleep quality and lack of sleep further exacerbates pain [23,24].

As far as we know, there are no data available at this moment regarding the impact of the COVID-19 pandemic and home lockdown measures on people with chronic pain. Therefore, we conducted a preliminary exploratory study to address this important issue. Our intention was to provide a general perspective, basically descriptive, from a cross-sectional online data survey, contextualized in one of the most affected countries worldwide: Spain. Specifically, we wanted to know how people with chronic pain felt in relation to different important domains, to analyze changes in their general health, and to explore changes in the coping strategies they have used and in their pain itself.

## 2. Methods

A cross-sectional online survey methodology was used. The Universitat Oberta de Catalunya’s Ethics Board evaluated and approved the study protocol on 22 April 2020.

### 2.1. Procedure

An online survey was created and implemented with the widely used online software Qualtrics (Qualtrics LLC, Provo, UT, USA). The survey was developed by the research group taking into account previous relevant literature about chronic pain prevalence and impact, available measures, and core outcome domains for chronic pain [25]. An important point was to make it as brief as possible to increase the response rate.

Once the research group reached a consensus regarding the survey content, it was sent to a panel of experts for review. At this stage, the questionnaire was fully reviewed by a methodologist with experience in health promotion, a researcher with experience in e-health and survey design, a psychologist with experience in health promotion research, a psychologist with experience in chronic pain management and research, and two psychologists with experience in clinical and health psychology research. Two people with chronic pain were also asked to answer the survey. If considered appropriate, their comments were included and the final survey was ready for dissemination. The online survey included a consent to participate in the study as well as statements on data protection laws. Below we describe the battery of variables gathered by this online survey.

The survey was open from 27 April to 25 May 2020. Spain was in a state of emergency from 13 March to 21 June, with restrictions placed on people’s movement. During this period, the Spanish population was required to stay at home, although different steps were taken until the country reached the so-called “new normality”. In this vein, for example, from 4 May people were allowed to exercise outside and walk with children, and from 11 to 25 May restaurants opened with some restrictions and only in some places. Therefore, there was high variability depending, basically, on the region where people resided and their work situation. In any case, all Spanish people had restrictions and their movement was limited during the emergency period. To make a clear point of reference for respondents, we asked them about the period since the lockdown started, which was the same for the whole country (14 March). No compensation was given for completing the survey and the average estimated time for completion was approximately 15 to 20 min.

### 2.2. Participants

Potential participants were contacted through the researchers’ social media, massive electronic mailing, and by emailing the survey through a long list of patient and regional chronic pain associations and social media channels. The final sample consisted of 502 people. Inclusion criteria were: (1) adults (aged 18 or older); (2) Spanish residents; (3) persistent or chronic pain (>3 months) reported, with at least two pain episodes per week; and (4) completion of at least 80% of the online survey.

### 2.3. Measures

#### 2.3.1. Sociodemographic Variables and Contextual Variables

We gathered sociodemographic data on age (in ranges of 10 years) and sex. We used multiple choice questions for civil status and education. We included other sociodemographic contextual variables in the survey that we hypothesized could affect the home lockdown situation: number of people living in the home, people in a situation of dependence living in the home, and number of people under 18 or above 65 years old living in the home. We also collected self-reported data about their personal COVID-19 infection status (positive, negative, unknown, COVID-like symptoms), infected people close to them and people close to them that had died due to COVID-19.

Job insecurity, or concern about the possibility of job loss, was measured through a 4-item scale developed by de Witte [26]. An example item is: “Chances are I will soon lose my job.” Participants give their responses on a 5-point Likert scale ranging from 1 (strongly disagree) to 5 (strongly agree). Cronbach’s alpha for this measure in our sample was 0.91. Worries about the future were measured through the 9-item scale developed by Höge et al. [27]. This measure was composed of three dimensions: labor, material and social worries. Labor worries reflected concerns about not maintaining their work conditions in the future and interference in their professional career (e.g., “I am worried about my future job conditions.”). Material worries addressed worries about reduced income in the future and having fewer possibilities to satisfy material needs and wishes (e.g., “I am worried about a future decline in my income.”). Social worries reflected worries about not maintaining and nurturing satisfactory family lives, partnerships, friendships, and other social contacts (e.g., “I am worried about not being able to have a happy family life or partnership in the future.”). Responses ranged from 1 (strongly disagree) to 5 (strongly agree). Cronbach’s alphas in our sample were 0.87 for labor worries, 0.87 for material worries and 0.77 for social worries.

#### 2.3.2. Description of Participants’ Pain Characteristics

We asked subjects to indicate how many months had passed since the onset of their chronic/persistent pain and what type of chronic pain they had, using a multiple-choice question as per the proposal by Treede et al. [28] for the classification of chronic pain for the ICD-11 diagnosis system. Therefore, the following entities were included: chronic primary pain, chronic cancer pain, chronic postsurgical and post-traumatic pain, chronic neuropathic pain, chronic headache and orofacial pain, chronic visceral pain and chronic musculoskeletal pain. A figure of the human body was used for gathering information about pain location and also for the location of the most bothersome pain problem. Pain frequency was measured by asking subjects about their usual frequency of pain through a multiple-choice question based on the work by Breivik et al. [29].

Besides these previous questions, we used the Spanish version of the Chronic Pain Grade Questionnaire [30], an 8-item measure widely used in epidemiological studies. It typically comprises a first item asking for pain frequency during the last six months and seven additional items in a 11-point Likert scale (from 0 = “no pain” to 10 = “the worst pain imaginable”) asking for patient’s status during the last three months. These items allow the calculation of (a) the characteristic pain intensity index (CPGQ-P; including items of worst and mean pain intensity during the last three months and current pain intensity; 0–30 range score); and (b) a disability score (CPGQ-D; with items of mean of interference in performing daily tasks, activities of daily life, work, and social occupations; 0–40 range score). The time frame of the questionnaire was slightly adapted in our study, since subjects were asked to rate their situation since the beginning of the home lockdown situation. According to Ferrer-Peña et al. [30], the Spanish version of the CPGQ shows adequate internal consistency (α = 0.87).

#### 2.3.3. Changes in Pain and Pain-Related Outcomes Associated with Lockdown

Taking the IMMPACT (Initiative on Methods, Measurement, and Pain Assessment in Clinical Trials) assessment domains [25] and the CPGQ as references, we designed a short questionnaire to assess the perceived changes in respondents’ situation since the beginning of home lockdown in comparison to their situation pre-lockdown. We asked them to rate their status on a scale from −10 (it has decreased extremely) to 10 (it has increased extremely), with 0 meaning no changes, for the following variables: pain intensity, frequency of pain episodes, pain interference in everyday activities, pain interference on work capacity (including homework), pain interference on hobbies, pain interference on social and familiar activities, distress caused by pain, support received from others when they were in pain, effects of pain on sleep and on physical activity.

#### 2.3.4. Changes in Functional Domains

The Patient Global Impression of Change (PGIC) and the Pain-Specific Impression of Change (PSIC) instruments [31] were used as self-reporting measures of perception of global change and change in specific domains. These measures are frequently used as indicators of patient-meaningful change in interventions for chronic pain and are scored on a 7-point Likert scale (from 1 = “much better” to 7 = “much worse”). Regarding specific domains evaluated in the PSIC, perceived changes in physical and social functioning, work-related activities, mood, and pain intensity were collected. In total, the six items from the two questionnaires were administered and adapted for asking about changes since the beginning of lockdown.

#### 2.3.5. Pain-Related Coping Strategies and Triggers

We asked participants with a single yes/no question if they had changed their pain coping strategies during lockdown. If they responded affirmatively, they were asked to inform if they had incorporated or stopped using the following strategies (they also had the option of choosing that they had neither incorporated nor stopped using them): increase of pain-related medication intake, stretching, physical exercise, resting, social support seeking, online health information seeking, and drinking.

Finally, we included a list of perceived pain triggers, and participants were asked to report whether each one had prompted pain episodes (Yes/No/Not sure) before and during lockdown. Perceived triggers included: stress, familiar or social conflicts, inadequate diet, uncertainty or worries about the future, sleep problems, feelings of insecurity, negative thoughts, feelings of sadness, feelings of loneliness, work-related factors, sedentarism, weather changes and fear of being infected with coronavirus.

### 2.4. Data Analysis

We computed descriptive statistics for each of the variables measured. We also tested if reported changes in pain and pain consequences were related to sociodemographic variables and lockdown background variables. We used Pearson correlations for continuous variables (e.g., relationship between CPGQ-P scores and changes in usual pain intensity) and a *t*-test for dichotomic variables (e.g., for testing differences between those participants who are and are not living with someone in dependence regarding changes in frequency of pain episodes). The same procedure was carried out to test the relationships with the changes in global domains assessed by the PSIC and the PGIC.

Apart from descriptive statistics, we tested triggers using McNemar’s test if there were significant differences between the percentage of people who informed of each specific variable as a trigger before and during the lockdown period. All the tests used were bilateral and the significance level was set at α < 0.05. All analyses were performed using IBM SPSS statistical software version 20.0 (IBM, Armonk, NY, USA).

## 3. Results

### 3.1. Sociodemographic, Contextual Variables and Job Perceptions

A total of 998 people visited our online survey, confirmed that they fulfilled the inclusion criteria, and agreed to participate, but 232 did not answer a single question. Of the remaining 766 subjects, 663 confirmed their residence in Spain with a multiple choice question. Of those, 536 were confirmed to meet the pain-related inclusion criteria through a question designed for this purpose. In the end, a total of 502 subjects completed at least 80% of the survey (most of them, 94.2%, completed the entire survey) and comprised the final sample. Most respondents were women (88%), between 30 and 59 years old (80.7%). Most were married (66.9%) and had reached secondary education or higher (87.8%). Socio-demographic data are displayed in Table 1.

Regarding contextual variables, 10.2% of the participants defined themselves as individuals in a situation of dependence, and 8% stated they were living together with someone in a status of dependence during lockdown. A total of 70.5% were not cohabitating with people under 18 during the lockdown. Of those cohabitating with children or adolescents, most were living with one or two (20.3% and 8.6%, respectively). The mean number of people living at home during the lockdown was 2.60 (standard deviation (SD): 1.06; range 1–7).

Finally, in relation to labor concerns (considering only those subjects of the sample who were working; *n* = 274), means were as follows: 2.60 (SD: 1.21) for job insecurity, 3.63 (SD: 1.21) for labor worries, 3.61 (SD: 1.15) for economic worries, and 3.02 (SD: 1.24) for personal worries.

In relation to COVID-19 infection, 1.6% of the sample had been infected, and 30.5% stated that they were not sure if they had been infected. A further 24.1% reported having someone close to them being infected and 16.9% reported that they were not sure if someone close to them had been infected by COVID-19. Finally, 13.5% reported having someone close to them die due to COVID-19.

### 3.2. Participant’s Pain Characteristics

Most of the participants (87.6%) reported pain in more than one pain location (mean pain locations: 5.01; SD: 3.18), with the abdomen, lower back and neck being the most frequently reported locations. The majority of the sample reported pain with a frequency of several times per week or more (93.2%). Mean pain duration was around seven years (79.33 months; SD = 90.73). See Table 1 for more details about specific diagnoses selected, most bothersome pain locations and pain frequency.

Mean for characteristic pain intensity since the beginning of lockdown assessed with CPGQ was 68.1 (SD = 17.62); for the disability score, the mean was 60 (SD = 25.43).

### 3.3. Changes in Pain and Pain-Related Outcomes Associated with Lockdown

Mean scores of all the scales are presented in Table 2, which also displays the frequency of participants who obtained a score less than 0 (indicating an improvement), a score of 0 (no change) or a score greater than 0 (indicating a worsening). As can be seen, most of the participants experienced worsening or no changes in pain and pain-related outcomes. Domains with more people reporting a worsening (and with higher mean scores), around 80%, were distress, sleep and interference of pain in physical activities. On the other hand, the area with a higher proportion of people reporting no changes or even an improvement was “support received from others”.

Correlations between each domain (changes in pain and pain-related outcomes) with CPGQ scores, age, duration of pain, labor perceptions and number of people living at home are also displayed in Table 2. There were significant positive correlations between CPGQ-P and CPGQ-D and each pain-related outcome. Pain duration was negatively and significantly correlated with changes in perceived social support (greater duration was related with greater diminution in the support received). Job perceptions were positively and significantly related with all domains except for support received. Age was not correlated with any of the domains. Number of people cohabiting was significantly related to greater distress caused by pain, greater effects on sleep, and increase in perceived social support, although the correlations were of small magnitude.

Regarding the *t*-tests, cohabitating with someone in a situation of dependence, gender and having someone close to you infected by coronavirus did not have a significant impact on any of the domains. Those who indicated having had someone close to them die due to COVID-19 reported a significantly greater effect of pain on physical activity (mean of 6.1 and 4.95, respectively; *t* = 2.07; *p* = 0.04). Those who were not sure of being infected by COVID-19 had significantly greater trouble sleeping than those who claimed not to have been infected (mean of 5.39 and 4.43, respectively; *t* = −2.26; *p* = 0.02). We could not compare those who reported being infected with those who did not or those who were unsure due to sample size limitations (only 8 participants confirmed that they had been infected).

### 3.4. Changes in Functional Domains

Descriptive statistics for the PSIC domains and global changes in the PGIC are presented in Table 3. Scores for the 6 items were significantly and positively correlated with the CPGQ-P, CPGQ-D, and with labor perceptions (the exception being the correlation between material worries and social activities). As can be seen in Table 3, the lowest correlations were between the CPGQ scores and changes in social activities, and the highest, between CPGQ scores and global well-being. In relation to labor perceptions, the highest correlations were with emotional state. Pain duration was not related to any of the 6 items. Number of people cohabiting was only significantly related with emotional state. Age was significantly related to small positive correlations with changes in physical capacity and labor and social activities.

Those living with someone in a situation of dependence had significantly worse outcomes in general health since the lockdown (mean of 5.41 and 4.95, respectively; *t* = −2.13; *p* = 0.03), physical capacity (mean of 5.54 and 5.12, respectively; *t* = −1.97; *p* < 0.05), and social activities (mean of 5.56 and 5.15, respectively; *t* = −2.01; *p* < 0.05). Those who were not sure of COVID-19 infection had worse outcomes compared to those who claimed not to be infected regarding changes in global well-being (mean of 5.17 and 4.91, respectively; *t* = −2.06; *p* = 0.04) and in changes in emotional state since the lockdown (mean of 5.58 and 4.16, respectively; *t* = −3.36; *p* = 0.001). Having someone close to them infected with COVID-19 or dying because of it and gender did not have a significant impact on any of the assessed domains.

### 3.5. Changes in Pain-Coping Strategies

More than half the sample reported having experienced changes in their way of coping with pain (54.5%). Table 4 displays the proportion of these people who incorporated, refrained from using, or neither incorporated nor dismissed each of the proposed coping strategies. As can be seen in Table 4, a substantial proportion of the participants incorporated new coping strategies during lockdown, with resting (54.5%), stretching (48.2%) and increase in medication intake (46.7%) being the most usually incorporated.

### 3.6. Changes in Perceived Pain Triggers

Frequency of perceived pain triggers before and during lockdown can be seen in Table 5. Stress was the trigger most frequently reported before lockdown, followed by weather changes and sleep problems. During lockdown, there was a significant increase in the proportion of participants who thought that the following variables triggered pain: worries about the future, sleep problems, feelings of insecurity, negative thoughts, sadness, loneliness, sedentarism, and fear of suffering from COVID−19. In contrast, the proportion of participants indicating that stress triggered pain was reduced during lockdown significantly.

## 4. Discussion

Our study sample was predominantly made up of women (88%) who typically suffered pain in more than one location and had longstanding problems. These characteristics are similar to those found in epidemiological studies. However, although a higher percentage of women is usual, it is not usually as high as it is in our study. For example, the proportion of women in one recent epidemiological study in Italy was 67% [32]. Our major proportion of women may be due to the fact that we sent information about our study to pain associations and groups via social media in which women tend to participate more frequently [33]. Available literature also confirms that women are more likely to participate in online surveys [34].

Characteristic pain intensity and disability scores from the beginning of lockdown assessed with the CPGQ were in the upper range (mean of 68.1 and 60, respectively). Although comparison between studies is quite complicated, our data suggest worse scores on both scales than those reported in other studies using the same questionnaire and reporting scores for the two scales [35]. Changes in pain and pain-related outcomes since the beginning of lockdown were correlated with pain intensity and disability scores and confirmed a worsening in all assessed domains (except for support received from others, which increased). There was an increase in pain intensity, frequency of pain episodes, pain interference (in everyday activities, work capacity, and leisure activities), distress caused by pain, and effects of pain on sleep and on physical activity. Higher mean scores (suggesting greater worsening) were found for distress, quality of sleep and physical activity. Similar results were found for PSIC domains and global changes. Specifically, when respondents were asked about changes (not necessarily related to pain) mean scores showed a worsening in physical activity, social activities, labor activities, emotional state and global well-being (mean scores were quite similar, around 5, for all the scales). These data highlight the importance of paying attention to people with chronic pain during health crises, since their pain problem and general health can worsen, as has been pointed out by experts [13]. Moreover contextual variables such as job insecurity, worries about the future, and number of people cohabiting are related with changes in pain and pain-related outcomes, and with global changes. Having someone close to you died because of COVID-19 and not being sure whether you were infected with the virus were also related with worse outcomes. Along the same lines, a recent study on the general population reported that a greater psychological effect was related to those with self-reported symptoms of COVID-19, with changes in employment activity [36] and with those cohabiting with two to four people (vs. those living alone or cohabiting with one person). In any case, here we consider longitudinal studies essential for studying causal relationships, building sound statistical models and truly understanding the experience of those people suffering from chronic pain. However, these preliminary data suggest the importance of taking into account the context in which people with chronic pain are involved during crises such as that caused by COVID-19.

More than half the participants reported changes in their way of managing their pain as a consequence of the pandemic. Of those, more than half reported having incorporated resting and around half informed of having increased their intake of medication. Resting can be a dangerous coping strategy if used permanently, as it can increase disability [37]. Medication intake is also something to take into account, requiring future research to explore whether people with chronic pain increase intake with or without a professional prescription. This is because medication patterns (particularly with prescription drugs such as opioids) require strict supervision by health professionals, and overdose of non-prescription analgesics represents a non-negligible risk [38]. In contrast, a good point is that nearly half the participants reported having started to use stretching to cope with pain, and nearly one third started using exercise (although almost another one third stopped exercising to cope with pain, probably due to the impossibility of doing so from home). Literature widely supports the benefit of stretching and exercising for managing pain [39], and the mass media and relevant stakeholders (e.g., patients’ associations and health-related websites) likely helped during this period since the idea of being active at home was widespread. For example, the Spanish Society for Pain’s website [40] encouraged people with chronic pain to stay active and exercise. It is also noteworthy that more than one third of the participants reported starting to use Internet resources to cope with pain. This is congruent with the fact that information and communication technologies (ICT) have great potential for facilitating access to pain management interventions [41], especially taking into account the difficulties in accessing pain management facilities as a consequence of the pandemic [11]. Considering the current health situation, it is probably an area in which society should invest more resources. It would also be interesting to work on the creation of online community networks, since people going through catastrophes need to feel they belong to a community [42].

An important contribution made by our work is the study of participants’ perceived triggers. This is because few studies have asked people with pain which factors they consider to trigger their pain [43,44,45,46]. It is interesting to explore whether people’s perceptions coincide with scientific literature and if these perceptions change as a consequence of the pandemic. Along these lines, stress was the most recognized factor as a trigger in our study prior to lockdown. As commented in the introduction, there is a lot of evidence of its role in pain literature [16,17]. However, it is curious that during the lockdown a slightly lower (yet significant) proportion of the participants chose stress as a pain trigger. This is probably because other triggers gained representativeness during the pandemic. Along these lines, there was a significant increase in people who stated that triggers related to cognitions (i.e., worries about the future, fear of being infected by COVID-19, and negative thoughts) triggered pain during the lockdown. The proportion of people who stated that sadness and loneliness triggered pain also increased significantly. Cognitions and emotions are constantly interacting with each other and with pain, and their role is widely recognized and supported in scientific literature [18,19,20,47]. The situation of uncertainty and emergency likely magnified all these factors. Finally, also in relation with available literature [23,24] sleeping problems were also widely recognized as triggers and their role increased during lockdown (being the most frequently mentioned by participants as a trigger).

The main implications of our results can be found in Table 6.

Besides these important implications, we have to acknowledge some limitations of our work. First, although online assessment has proven to generate reliable data [48], it has inherent limitations, such as self-selection bias and sample representativeness [49]. For instance, there are difficult-to-reach people characterized by digital illiteracy. Second, we did not use a stratified random sampling technique, which may have an impact on the generalizability of our findings. Third, as commented, the design was not longitudinal, which prevents it from exploring causal models. Fourth, a more in-depth assessment (e.g., including interviews with people in pain) would have been very useful to obtain a more global picture. However, despite these limitations, our results are important, as they show a clear impact on pain and general well-being and highlight some factors (and triggers) that can be helpful for creating prevention programs or guidelines for health professionals. Firstly, among the measures to be implemented, it may be useful to consider those that have been indicated for the general population. These include the use of psychological first aid, after evaluating critical needs, and intervening early on stress [50]. E-health has emerged as a clear need and should be implemented for general health and pain interventions while lockdown and social distancing measures are in place [11,51]. Lastly, and more specifically, in future lockdown situations it would probably be useful to help people with chronic pain to curb uncertainty, provide online support communities, manage loneliness, and take care of sleeping habits.

## Figures and Tables

**Table 1:** Sociodemographic and pain characteristics of the study sample.

Variable	%
Age ranges	
18–29	7%
30–39	21.90%
40–49	31.30%
50–59	27.50%
60–69	10.40%
70–79	1.60%
80–89	0.40%
Marital status	
Married/Living with a partner	66.90%
Separated/Divorced	10.20%
Single	20.50%
Widowed	2.40%
Education level	
No studies	0.20%
Primary school	12%
Secondary school	40.40%
University	26.90%
Postgraduate studies	20.50%
Employment status	
Temporary employment	8.90%
Permanent employment	44.30%
Self-employed	7.80%
Unemployed but searching for a job	7.80%
Unemployed and not searching for a job	8%
Student	2.70%
Retired	7.60%
Others	12.90%
Type of pain *	
Primary pain	53.20%
Musculoskeletal pain	52.20%
Headache and orofacial pain	26.70%
Other	26.30%
Neuropathic pain	19.50%
Visceral pain	12.50%
Postsurgical/posttraumatic pain	10.40%
Cancer pain	1.60%
Most bothersome pain location	
Low back	18.10%
Abdomen	18.10%
Neck	17.30%
Buttocks	13.10%
Legs	10.40%
Head	7.80%
Upper back	4.80%
Shoulder	3%
Hands	2.40%
Arms	2.40%
Feet	2%
Chest	0.60%
Pain frequency	
Always	39.60%
Daily	36.10%
Several times per week	17.50%
Once per week **	1%
Several times per month	5%
Once per month	0.60%
Less than once per month	0.20%

* These were multiple choice questions; ** Inclusion criteria for the study were having chronic pain with a duration of at least three months and at least two episodes per week. All respondents indicated meeting these criteria, but some of them, in the question related to frequency, indicated a range lower than two times per week. We understand that they were probably undergoing a period of less frequency.

**Table 2:** Perceived changes in pain and pain-related outcomes, and correlations between study variables.

Descriptives			Correlations with Pain Characteristics		Correlations with Sociodemographic and Contextual Variables						
VARIABLE	Mean (SD)	Proportion (%) of Improvement, no Change and Worsening	CPGQ-P	CPGQ-D	Age	Pain Duration	Job Insecurity	Labor Worries	Material Worries	Social Worries	Number People Cohabitating
Usual pain intensity	3.4 (3.89)	9.3–19.9–70.8	0.43 *	0.40 *	0.04	0.04	0.16 ***	0.26 *	0.22 *	0.20 **	0.04
Frequency of pain episodes	3.55 (4)	8.7–19.7–71.6	0.42 *	0.41 *	0.05	0.01	0.13 ***	0.23 *	0.18 ***	0.21 **	0.007
Pain interference in everyday activities	3.47 (4.1)	8.5–22–69.5	0.48 *	0.50 *	0.07	0.002	0.18 ***	0.35 *	0.26 *	0.29 *	0.01
Pain interference on work capacity	3.76 (4.14)	7.1–20.1–72.8	0.48 *	0.53 *	0.07	−0.02	0.19 ***	0.34 *	0.26 *	0.28 *	0.04
Pain interference on leisure, social and familiar activities	3.41 (4.22)	8.1–22.8–69.1	0.48 *	0.54 *	0.04	−0.01	0.19 **	0.38 *	0.28 *	0.32 *	0.07
Distress caused by pain	5 (4.45)	6.7–13.2–80.1	0.43 *	0.45 *	−0.02	−0.04	0.15 ***	0.34 *	0.28 *	0.28 *	0.11 ***
Support received from others	2.56 (5.14)	55.7–29.5–14.8	0.15 **	0.16 *	0.05	−0.11 ***	0.03	0.04	0.08	0.03	0.13 ***
Effects on sleep	4.69 (4.37)	6.5–14.4–79.1	0.38 *	0.36 *	−0.06	0.03	0.19 ***	0.30 *	0.25 *	0.23 *	0.09 ***
Effects on physical activity	5.11 (4.26)	5.5–14.6–79.9	0.41 *	0.47 *	−0.02	−0.001	0.10	0.33 *	0.26 *	0.23 *	0.04

Note: * *p* < 0.0001; ** *p* < 0.001; *** *p* < 0.05; CPGQ-P = Characteristic Pain Intensity Index; CPGQ-D = Disability Score.

**Table 3:** Descriptives in Pain-Specific Impression of Change (PSIC) domains and global changes (Patient Global Impression of Change (PGIC)) and correlations between study measures.

Descriptives		Correlations with Pain Characteristics		Correlations with Sociodemographic and Contextual Variables						
VARIABLE	Mean (SD)	CPGQ-P	CPGQ-D	Age	Pain Duration	Job Insecurity	Labor Worries	Material Worries	Social Worries	Number People Cohabitating
Global well-being	4.99 (1.29)	0.43 *	0.4 *	0.05	0.004	0.18 **	0.29 *	0.22 **	0.22 *	0.06
Activity/physical capacity	5.16 (1.27)	0.27 *	0.30 *	0.09 ***	0.04	0.15 ***	0.26 *	0.19 **	0.24 *	−0.02
Social activities	5.18 (1.24)	0.14 **	0.19 *	0.11 ***	0.02	0.15 ***	0.18 **	0.08	0.21 **	0.02
Laboral activities	5 (1.25)	0.31 *	0.38 *	0.09 ***	−0.01	0.17 **	0.22 *	0.20 **	0.18 **	0.09
Emotional state	5.29 (1.32)	0.41 *	0.38 *	−0.007	−0.02	0.26 *	0.31 *	0.27 *	0.29 *	0.11 ***
Pain	5.14 (1.29)	0.47 *	0.42 *	0.05	0.06	0.15 ***	0.25 *	0.23 *	0.25 *	0.04

Note: * *p* < 0.0001; ** *p* < 0.005; *** *p* < 0.05; CPGQ-P = Characteristic Pain Intensity Index; CPGQ-D = Disability Score.

**Table 4:** Changes in coping strategies during lockdown.

Coping Strategy	Incorporated (%)	Dismissed (%)	Neither Incorporated nor Dismissed (%)
Resting	54.5	7.5	38.0
Stretching	48.2	9.0	42.7
Increase in medication intake	46.7	3.1	50.2
Exercising	32.1	28.6	39.3
Using internet resources	33.6	6.3	60.2
Social support	26.8	12.8	60.3
Alcohol consumption	7.8	11.4	80.8

**Table 5:** Changes in perceived pain triggers before and during lockdown.

	Before			During			
Trigger	Yes (%)	No (%)	Not Sure (%)	Yes (%)	No (%)	Not Sure (%)	McNemar’s Test *p* Value
Stress	83.5	8.2	8.2	76.3	13.5	10.1	<0.0001
Weather changes	67.7	21.1	11.2	68.5	20.7	10.8	0.62
Sleep problems	66.0	26.6	7.4	79.1	15.9	5.1	<0.0001
Working issues	60.3	30.0	9.7	57.5	33.8	8.7	0.18
Worries about the future	59.8	27.3	12.9	71.2	17.1	11.6	<0.0001
Familiar or social conflicts	58.8	30	11.2	60.7	30.0	9.3	0.36
Sedentarism	57.9	31.3	10.8	75.5	16.7	7.8	<0.0001
Sadness	55.0	33.0	12.1	68.1	21.1	10.8	<0.0001
Negative thoughts	49.5	35.7	14.8	61.5	26.0	12.5	<0.0001
Feelings of insecurity	48.2	37.4	14.4	62.6	25.2	12.3	<0.0001
Diet	40.2	36.4	23.5	43.6	33.8	22.6	0.06
Loneliness	37.2	50.1	12.7	45.2	42.3	12.5	<0.0001
Fear of suffering from COVID-19	20.3	67.9	11.8	39.1	48.2	12.7	<0.0001

Note: respondents who said “No” or “Not Sure” were grouped together to perform McNemar’s test.

**Table 6:** Main implications of the results.

Lockdown seems to be related to a worsening in pain, pain-related domains and global changes. Management of pain was negatively affected during the lockdown for many people who tended to use resting and increased medication consumption; however, as a positive effect, some people started stretching and exercising for pain. Worries about the future, sleep problems, feelings of insecurity, negative thoughts, sadness, loneliness, sedentarism, and fear of suffering from COVID-19 gained representativeness as triggers during the lockdown. Particular attention should be given to those with chronic pain problems in emergency healthcare situations; and eHealth probably has the power to maintain care for vulnerable populations, such as people with chronic pain during global healthcare emergencies.

## References

[1] World Health Organization WHO Coronavirus Disease (COVID-19) Dashboard. https://covid19.who.int.

[2] Wang C., Pan R., Wan X., Tan Y., Xu L., Ho C.S., Ho R.C. (2020). Immediate Psychological Responses and Associated Factors during the Initial Stage of the 2019 Coronavirus Disease (COVID-19) Epidemic among the General Population in China. Int. J. Environ. Res. Public Health.

[3] Huarcaya J. (2020). Consideraciones sobre la salud mental en la pandemia de Covid-19. Rev. Peru. Med. Exp. Salud Pública.

[4] Gunnell D., Appleby L., Arensman E., Hawton K., John A., Kapur N., Khan M., O’Connor R.C., Pirkis J., Caine E.D. (2020). Suicide risk and prevention during the COVID-19 pandemic. Lancet Psychiatry.

[5] Hawryluck L., Gold W.L., Robinson S., Pogorski S., Galea S., Styra R. (2004). SARS Control and Psychological Effects of Quarantine, Toronto, Canada. Emerg. Infect. Dis..

[6] Eurofound Living, Working and COVID-19 First Findings—April 2020. https://www.eurofound.europa.eu/publications/report/2020/living-working-and-covid-19-first-findings-april-2020.

[7] International Labor Organization Almost 25 Million Jobs Could Be Lost Worldwide as a Result of COVID-19, Says ILO. https://www.ilo.org/global/about-the-ilo/newsroom/news/WCMS_738742/lang--en/index.htm.

[8] Coibion O., Gorodnichenko Y., Weber M. (2020). Labor Markets during the COVID-19 Crisis: A Preliminary View. SSRN Electron. J..

[9] Meltzer H., Bebbington P., Brugha T., Jenkins R., McManus S., Stansfeld S. (2009). Job insecurity, socio-economic circumstances and depression. Psychol. Med..

[10] Romeyke T., Noehammer E., Stummer H. (2020). COVID-19 Case Report: An 84-Year-Old Man with Exacerbation of Multiple Comorbidities Due to COVID-19 Managed by a Multidisciplinary Team Using Patient-Reported Outcomes. Am. J. Case Rep..

[11] Eccleston C., Blyth F.M., Dear B.F., Fisher E.A., Keefe F.J., Lynch M.E., Palermo T.M., Reid M.C., Williams A.C.D.C. (2020). Managing patients with chronic pain during the COVID-19 outbreak. Pain.

[12] Murray C.J., Atkinson C., Bhalla K., Birbeck G., Burstein R., Chou D., Murray U.S. (2013). The State of US Health, 1990-2016: Burden of Diseases, Injuries, and Risk Factors among US States. JAMA.

[13] El-Tallawy S.N., Nalamasu R., Pergolizzi J.V., Gharibo C. (2020). Pain Management during the COVID-19 Pandemic. Pain Ther..

[14] Shanthanna H., Strand N.H., Provenzano D.A., Lobo C.A., Eldabe S., Bhatia A., Wegener J., Curtis K., Cohen S.P., Narouze S. (2020). Caring for patients with pain during the COVID-19 pandemic: Consensus recommendations from an international expert panel. Anaesthesia.

[15] Burke N.N., Finn D.P., McGuire B.E., Roche M. (2016). Psychological stress in early life as a predisposing factor for the development of chronic pain: Clinical and preclinical evidence and neurobiological mechanisms. J. Neurosci. Res..

[16] Crettaz B., Marziniak M., Willeke P., Young P., Hellhammer D., Stumpf A., Burgmer M. (2013). Stress-Induced Allodynia—Evidence of Increased Pain Sensitivity in Healthy Humans and Patients with Chronic Pain after Experimentally Induced Psychosocial Stress. PLoS ONE.

[17] Ortego G., Villafañe J.H., Doménech-García V., Berjano P., Bertozzi L., Herrero P. (2016). Is there a relationship between psychological stress or anxiety and chronic nonspecific neck-arm pain in adults? A systematic review and meta-analysis. J. Psychosom. Res..

[18] Aremka L.M., Andridge R.R., Fagundes C.P., Alfano C.M., Povoski S.P., Lipari A.M., Agnese D.M., Arnold M.W., Farrar W.B., Yee L.D. (2014). Pain, depression, and fatigue: Loneliness as a longitudinal risk factor. Health Psychol..

[19] Smith T.O. (2017). “On their own”: Social isolation, loneliness and chronic musculoskeletal pain in older adults. Qual. Ageing Older Adults.

[20] Smith T.O., Dainty J.R., Williamson E., Martin K.R. (2018). Association between musculoskeletal pain with social isolation and loneliness: Analysis of the English Longitudinal Study of Ageing. Br. J. Pain.

[21] Booth J., Moseley G.L., Schiltenwolf M., Cashin A., Davies M., Hübscher M. (2017). Exercise for chronic musculoskeletal pain: A biopsychosocial approach. Musculoskelet. Care.

[22] Voitsidis P., Gliatas I., Bairachtari V., Papadopoulou K., Papageorgiou G., Parlapani E., Syngelakis M., Holeva V., Diakogiannis I. (2020). Insomnia during the COVID-19 pandemic in a Greek population. Psychiatry Res..

[23] Mills S.E., Nicolson K.P., Smith B.H. (2019). Chronic pain: A review of its epidemiology and associated factors in population-based studies. Br. J. Anaesth..

[24] Smith M.T., A Haythornthwaite J.A. (2004). How do sleep disturbance and chronic pain inter-relate? Insights from the longitudinal and cognitive-behavioral clinical trials literature. Sleep Med. Rev..

[25] Turk D.C., Dworkin R.H., Allen R.R., Bellamy N., Brandenburg N., Carr D.B., Cleeland C., Dionne R., Farrar J.T., Galer B.S. (2003). Core outcome domains for chronic pain clinical trials: IMMPACT recommendations. Pain.

[26] De Witte H. (1992). Langdurig Werkozen: Tussen Optimisten en Teruggetrokkenen.

[27] Höge T., Sora B., Weber W.G., Peiró J.M., Caballer A. (2015). Job insecurity, worries about the future, and somatic complaints in two economic and cultural contexts: A study in Spain and Austria. Int. J. Stress Manag..

[28] Treede R.-D., Rief W., Barke A., Aziz Q., Bennett M.I., Benoliel R., Cohen M., Evers S., Finnerup N.B., First M.B. (2015). A classification of chronic pain for ICD-11. Pain.

[29] Breivik H., Collett B., Ventafridda V., Cohen R., Gallacher D. (2006). Survey of chronic pain in Europe: Prevalence, impact on daily life, and treatment. Eur. J. Pain.

[30] Ferrer-Peña R., Gil Martinez A., Pardo-Montero J., Jiménez-Penick V., Gallego-Izquierdo T., La Touche R. (2016). Adaptación y validación de la Escala de gradación del dolor crónico al español. Reumatol. Clín..

[31] Scott W., McCracken L.M. (2015). Patients’ Impression of Change Following Treatment for Chronic Pain: Global, Specific, a Single Dimension, or Many?. J. Pain.

[32] Latina R., De Marinis M.G., Giordano F., Osborn J.F., Giannarelli D., Di Biagio E., Varrassi G., Sansoni J., Bertini L., Baglio G. (2019). Epidemiology of Chronic Pain in the Latium Region, Italy: A Cross-Sectional Study on the Clinical Characteristics of Patients Attending Pain Clinics. Pain Manag. Nurs..

[33] Chen G.M. (2013). Why do women bloggers use social media? Recreation and information motivations outweigh engagement motivations. New Media Soc..

[34] Blasius J., Brandt M. (2010). Representativeness in Online Surveys through Stratified Samples. Bull. Sociol. Methodol..

[35] Salaffi F., Stancati A., Grassi W. (2006). Reliability and validity of the Italian version of the Chronic Pain Grade questionnaire in patients with musculoskeletal disorders. Clin. Rheumatol..

[36] Odriozola-González P., Planchuelo-Gómez Á., Irurtia M.J., De Luis-García R. (2020). Psychological effects of the COVID-19 outbreak and lockdown among students and workers of a Spanish university. Psychiatry Res..

[37] Foster N.E., Anema J.R., Cherkin D., Chou R., Cohen S.P., Gross D.P., Ferreira P., Fritz J.M., Koes B., Peul W. (2018). Prevention and treatment of low back pain: Evidence, challenges, and promising directions. Lancet.

[38] Wolf M.S., King J., Jacobson K., Di Francesco L., Bailey S.C., Mullen R., McCarthy D., Serper M., Davis T.C., Parker R.M. (2012). Risk of Unintentional Overdose with Non-Prescription Acetaminophen Products. J. Gen. Intern. Med..

[39] Kroll H.R. (2015). Exercise Therapy for Chronic Pain. Phys. Med. Rehabil. Clin. N. Am..

[40] Sociedad Epañola del Dolor Información y Recomendaciones de la SED para Pacientes con Dolor Crónico ante la Pandemia por Covid-19. https://www.sedolor.es/infirmacion-y-recomendaciones-de-la-sed-para-pacientes-con-dolor-cronico-ante-la-pandemia-por-covid-19/.

[41] Nieto R. (2014). Secondary prevention of chronic pain: Can internet help?. Pain Manag..

[42] Reissman D.B., Watson P.J., Klomp R.W., Tanielian T.L., Prior S.A. (2006). Pandemic Influenza Preparedness: Adaptive Responses to an Evolving Challenge. J. Homel. Secur. Emerg. Manag..

[43] Crowe M., Whitehead L., Gagan M.J., Baxter G.D., Panckhurst A. (2010). Self-management and chronic low back pain: A qualitative study. J. Adv. Nurs..

[44] Kawi J. (2014). Chronic low back pain patients’ perceptions on self-management, self-management support, and functional ability. Pain Manag. Nurs..

[45] Roth-Isigkeit A. (2005). Pain among Children and Adolescents: Restrictions in Daily Living and Triggering Factors. Pediatrics.

[46] Setchell J., Costa N., Ferreira M., Hodges P.W. (2019). What decreases low back pain? A qualitative study of patient perspectives. Scand. J. Pain.

[47] Martinez-Calderon J., Jensen M.P., Morales-Asencio J.M., Luque-Suarez A. (2019). Pain Catastrophizing and Function In Individuals with Chronic Musculoskeletal Pain. Clin. J. Pain.

[48] Gwaltney C.J., Shields A.L., Shiffman S. (2008). Equivalence of Electronic and Paper-and-Pencil Administration of Patient-Reported Outcome Measures: A Meta-Analytic Review. Value Health.

[49] Wright K.B. (2006). Researching Internet-Based Populations: Advantages and Disadvantages of Online Survey Research, Online Questionnaire Authoring Software Packages, and Web Survey Services. J. Comput. Commun..

[50] Ramírez-Ortiz J., Castro-Quintero D., Lerma-Córdoba C., Yela-Ceballos F., Escobar-Córdoba F. Consecuencias de la Pandemia COVID-19 en la Salud Mental Asociadas al Aislamiento Social. https://preprints.scielo.org/index.php/scielo/preprint/view/303.

[51] Tull M.T., Edmonds K.A., Scamaldo K.M., Richmond J.R., Rose J.P., Gratz K.L. (2020). Psychological Outcomes Associated with Stay-at-Home Orders and the Perceived Impact of COVID-19 on Daily Life. Psychiatry Res..

